# Hierarchical cluster analysis of immunophenotype classify AML patients with NPM1 gene mutation into two groups with distinct prognosis

**DOI:** 10.1186/1471-2407-13-107

**Published:** 2013-03-08

**Authors:** Chien-Yuan Chen, Wen-Chien Chou, Woei Tsay, Jih-Luh Tang, Ming Yao, Sheng-Yi Huang, Hwei-Fang Tien

**Affiliations:** 1 Departments of Internal Medicine, National Taiwan University Hospital, No. 7, Chung-Shan South Road, Taipei 100, Taiwan; 2 Laboratory Medicine, National Taiwan University Hospital, No. 7, Chung-Shan South Road, Taipei, 100, Taiwan

**Keywords:** Acute myeloid leukemia, NPM1 mutation, Immunophenotype, Prognosis

## Abstract

**Background:**

The prognostic implication of immunophenotyping in acute myeloid leukemia (AML) patients with *NPM1* mutation remains unclear.

**Methods:**

Ninety-four of 543 AML patients diagnosed with *NPM1* mutation between 1987 and 2007 were studied. The expression of surface antigens on leukemic cells was evaluated with respect to clinical manifestations and outcomes. In order to validate the prognostic effect of the immunophenotypic cluster, another 36 patients with *NPM1* mutation diagnosed between 2008 and 2010 were analyzed.

**Results:**

Ninety-four patients with *NPM1* mutations and complete immunophenotyping data were enrolled for a hierarchical cluster analysis and the result was correlated with clinico-laboratory characteristics. Clustering analysis divided the patients with *NPM1* mutations into the following two groups: group I, CD34(−)/CD7(−), but with variable expression of HLA-DR; and group II, HLA DR(+)/CD34(+)/CD7(+). With a median follow-up of 53 months, the group II patients had a significantly shorter relapse-free survival (RFS, median: 3 vs. 23 months, p = 0.006) and overall survival (OS, median: 11 vs. 40 months, p = 0.02) than group I patients. Multivariate analysis of variables, including clinico-laboratory data and other gene mutations revealed that the immunophenotypic cluster is an independent prognostic factor (RFS, p = 0.002; OS, p = 0.024). In order to confirm the prognostic effect of the immunophenotypic cluster, another 36 patients with *NPM1* mutation diagnosed between 2008 and 2010 were validated. Hierarchical cluster analysis also showed two distinct clusters, group I patient showed significant better RFS (*p* = 0.021), and OS (*p* = 0.055). In total, we stratified 130 *NPM1*-mutant patients, by *FLT3-ITD* mutation and immunophenotypic cluster into distinct prognostic groups (RFS, p < 0.001 and OS, p = 0.017).

**Conclusions:**

Among *NPM1*-mutated AML, the antigen expression pattern of HLADR(+) CD34(+) CD7(+) is associated with a poor prognosis, independent to the *FLT3-ITD* mutation.

## Background

Acute myeloid leukemia (AML) is a heterogeneous group of diseases characterized by increasing immature progenitors in the bone marrow and peripheral blood. The leukemic subtypes are crucial to treatment and prognosis. Immunophenotyping by flow cytometry has been extensively used for the diagnosis and classification of acute leukemia [1,2]. Detection of leukemia-associated immunophenotypes by flow cytometry is also recognized as an important tool in monitoring minimal residual disease and predicting clinical outcome [3-5]. Karyotype is another important prognostic factor by which AML patients can be stratified into good-, intermediate-, and poor-risk groups. Leukemic blasts in AML with recurrent cytogenetic abnormalities, such as t(8;21), t(15;17), and inv(16) show specific antigen expression patterns [6]. Recently, an association of *CEBPA* mutation with a distinct immunophenotype was also reported [7].

About 40%-50% of AML patients have a normal karyotype of leukemic cells, and one-half of these patients have mutations of nucleophosmin (*NPM1)*[8,9], which encodes a shuttle protein transporting continuously between the nucleus and cytoplasm [10]. *NPM1* mutations are usually associated with absence of HLA-DR and CD34 expression [11]; however, the surface marker expression in blasts varies in individual AML patients and the clinical implication of immunophenotype in this subtype of AML remains unclear. Previous reports have suggested that expression of some surface antigens is correlated with clinical outcome in AML patients [12-14], but the prognostic significance of immunophenotype is still an issue of controversy [15,16]. Most studies analyzed the prognostic implication of individual antigens, and usually in a heterogeneous population of AML patients with various genetic abnormalities. In this study, we performed a hierarchical cluster analysis of the immunophenotype expression profiles in a relatively homogeneous cohort of AML patients with *NPM1* mutations, and correlated the results with clinical characteristics, other gene mutations, and prognoses.

## Methods

### Patients

Five hundred forty-three patients diagnosed as having *de novo* AML at the National Taiwan University Hospital between 1987 and 2007 were recruited in this study as the investigation cohort. In order to confirm the prognostic implication of the immunophenotypic profile, another 36 AML patients diagnosed with *NPM1* mutation between 2008 and 2010 were enrolled as the validation cohort. The informed consents were collected from all living patient. The NPM1 mutation was retrospectively checked in part of patients. Cryopreserved samples were collected from marrow bank according to the criteria of local ethics committee. This research conformed to the Helsinki Declaration and was approved by the National Taiwan University Hospital Research Ethics Committee.

### Immunophenotype

A panel of monoclonal antibodies, including HLADR, CD2, CD7, CD11b, CD13, CD14, CD15, CD19, CD33, CD34, CD41a, and CD56, was used to characterize the phenotypes of the leukemic cells as previously described [11].

### Cytogenetic analysis

Cytogenetic analysis was performed as described previously [17]. Briefly, the bone marrow and/or peripheral blood cells were harvested either directly or after 1–3 days of culture. Metaphase chromosomes were banded by the conventional trypsin-Giemsa banding technique and karyotyped according to ISCN [18].

### Gene mutation analysis

Mononuclear cells obtained from bone marrow aspirates were isolated by Ficoll-Hypaque gradient centrifugation and cryopreserved. Genomic DNAs were extracted and amplified by Illustra GenomiPhi V2 DNA amplification kit as described by the manufacturer (GE Healthcare). The primer design was according to the previous study [7,11,19-21]. Analysis of *NPM* exon 12 mutation was done as described by Falini et al. [8,11]. Briefly, the final volume for PCR reaction was 35 μL containing 200 ng DNA, 200 nmol/L deoxynucleotide triphosphate, 2 mmol/L MgSO_4_, 140 nmol/L of each primer, and 1 unit of AmpliTaq Gold polymerase (Applied Biosystems, Foster City, CA). PCR was done by heating at 95°C for 10 minutes, followed by 35 cycles of 95°C for 45 seconds, 49°C for 1 minute, and 72°C for 1 minute, with a final step for 10 minutes at 72°C. PCR products were electrophoresed on 2% agarose gels, purified and sequenced using the BigDye Terminator v3.1 Cycle Sequencing kit, which contained AmpliTaq DNA polymerase FS (Applied Biosystems), on an automated ABI-3100 Genetic Analyzer (Applied Biosystems). Abnormal sequencing results were confirmed by at least two repeated analyses. Analysis of the gene mutations of *CEBPA*[7]*, MLL-ITD*[19]*, WT1*[20]*, FLT3-ITD, FLT3-TKD, JAK2, PTPN11*, *NRAS,* and *KRAS*[21] was performed by polymerase chain reaction and direct sequencing. Abnormal sequencing results were confirmed by at least two repeated analyses.

### Statistics

Comparisons between groups were made with the ANOVA and chi-square tests. Hierarchical cluster analysis was performed with an agglomeration schedule, and the cluster distance was expressed as the Binary Square Euclidean distance [22,23]. A dendrogram was plotted using the average linkage method. Survival curves were plotted by the Kaplan-Meier method; differences between the curves were analyzed by the log-rank test. Multivariate Cox regression analysis was used to investigate independent prognostic factors for overall survival and relapse free survival. All statistical analyses were performed with SPSS 18.0 for Windows (SPSS, Inc., Chicago, IL, USA). Values of *P <* 0.05 were considered significant.

## Results

### Clinical characteristics of patients with *NPM1* gene mutations

The clinical and laboratory data of the 543 AML patients are shown in Table 1. There were 315 men and 228 women with a median age of 48 years; 52 patients were children less than 18 years and 491 were adults. *NPM1* gene mutations were detected in 108 (19.8%) of AML patients overall, and in 90 (37.5%) of the 241 AML patients with a normal karyotype, which were in agreement to our previous report [11]. *NPM1* gene mutations were rarely detected in children (2/52 (3.8%) in children vs. 106/491 (21.2%) in adults, p < 0.001). Females had a higher incidence of *NPM1* mutations than males (25.4% vs. 15.9%, p < 0.001). *NPM1* mutations were closely associated with HLA-DR(−), CD33(+), and CD34(−) (p < 0.001 for all three markers, Table 1).

**Table 1:** **Clinico-laboratory characteristics in AML patients with *NPM1 *mutations**

	**Total AML patients (n = 543)**	**AML patients with *NPM1 *mutation (n = 108)**	**AML patients with wild type *NPM1*(n = 435)**	**P-Value**
Age^#^				0.001
Adult(>18 years)	491	106(21.6)	385(78.4)	
Children	52	2(3.8)	50(96.2)	
Gender^#^				0.007
Male	315	50(15.9)	265(84.1)	
Female	228	58(25.4)	170(74.6)	
Laboratory data				
WBC(uL)	20660	38860	14260	0.002
Hemoglobin (g/dL)	8.1	8.4	8.0	0.095
Platelet(uL)	43000	52000	40000	0.865
LDH (units/L)	861	1068	826	0.149
FAB subtype^#^				0.008
M0	10	0(0)	10(100)	
M1	120	23(19.2)	97(80.8)	
M2	184	40(21.7)	144(78.3)	
M3	40	0(0)	40(100)	
M4	131	34(26.0)	97(74.0)	
M5	33	10(30.3)	23(69.7)	
M6	11	1(9.1)	10(90.9)	
M7	3	0(0)	3(100)	
Undetermined	5	0(0)	5(100)	
Cytogenetic^#^*				<0.001
Normal karyotype	241	90(37.3)	151(62.7)	
Abnormal karyotype	283	13(4.6)	270(95.4)	
Immunophenotype**				
HLA-DR	364/515	50/105(47.6)	314/410(76.6)	<0.001
CD7	100/511	16/104(15.4)	84/407(20.6)	0.269
CD13	481/519	96/105(91.4)	385/414(93.0)	0.536
CD14	70/503	18/104(17.3)	52/399(13.0)	0.267
CD15	237/511	46/104(44.2)	191/407(46.9)	0.660
CD33	472/518	105/105(100)	367/413(88.9)	<0.001
CD34	328/514	22/104(21.1)	306/410(74.6)	<0.001
CD56	109/457	19/95(20.0)	90/362(24.9)	0.347

*Only 524 patients had cytogenetic data.

** Number of patients with positive expression/number of patients studied.

^# ^Number of patients (%).

### Hierarchical cluster analysis of immunophenotypes in patients with *NPM1* gene mutations

**Figure 1:**
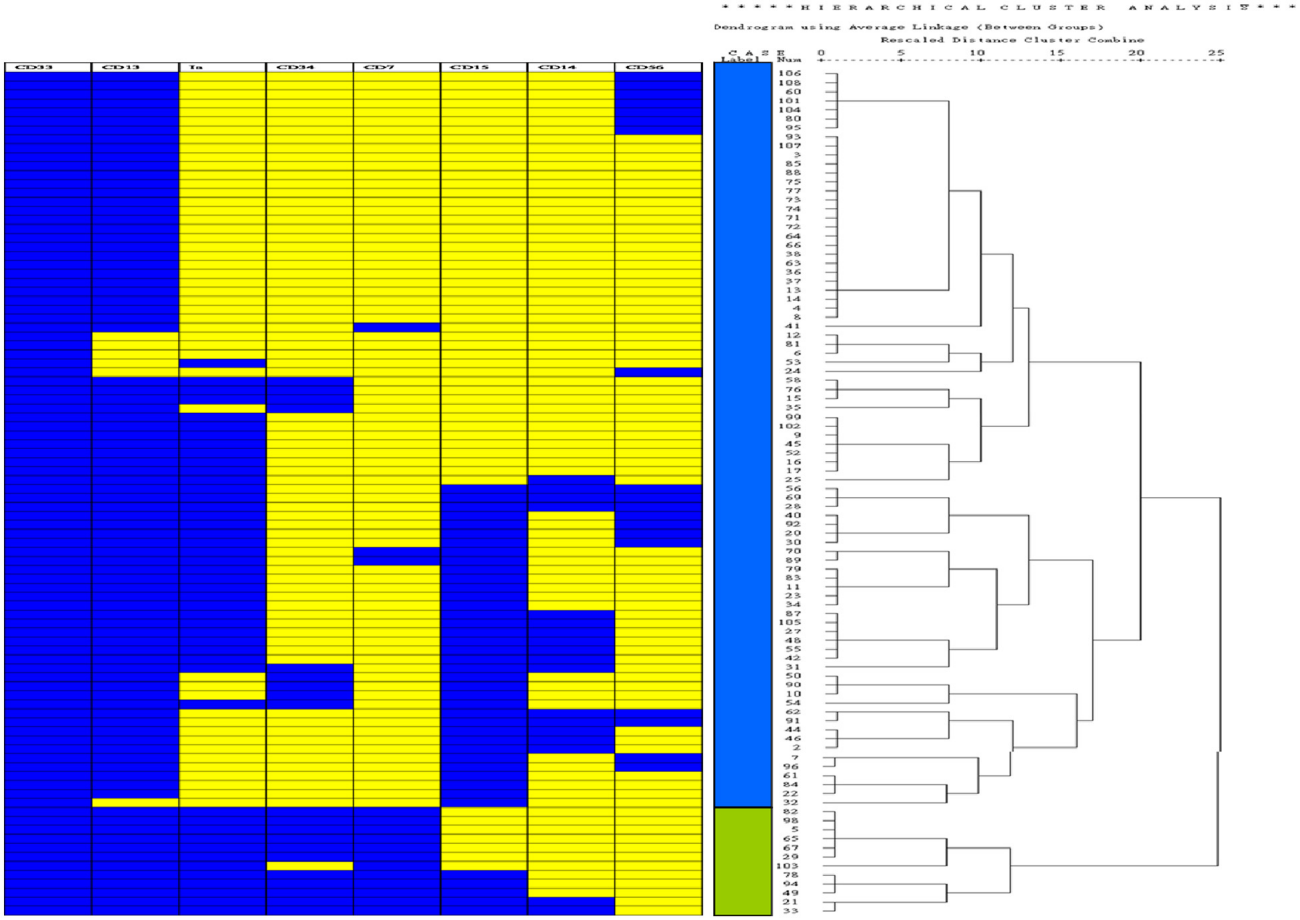
**Hierarchical cluster analysis of 94 *NPM1- *mutated patients.**

Hierarchical cluster analysis was performed based on the expression profile of 8 surface markers (HLADR, CD34, CD13, CD33, CD7, CD14, CD15, and CD56). The clustering result is displayed by the Treeview dendrogram (Figure 1). The rows represented individual cases and the columns were the results of expression of individual surface markers. The blue color indicates positive expression and the yellow color indicates negative expression. Ninety-four patients with *NPM1* mutations and complete immunophenotype data were enrolled in the hierarchical cluster analysis. The clustering analysis divided the patients with *NPM1* mutations into two groups, designated as group I (n = 82, 87%; blue color) and group II (n = 12, 13%; green color), based on the expression profiles of the immunophenotype (Figure 1). Leukemic cells from most group I patients were CD34(−)/CD7(−)/CD13(+)/CD33(+), but with variable expression of HLA DR and other antigens, while those from almost all group II patients were HLA DR(+)/CD34(+)/CD7(+)/CD13(+)/CD33(+); only one group II patients showed absence of CD34 on leukemic cells.

### Correlation of immunophenotypic cluster with clinico-laboratory features, associated gene mutations, and clinical outcome

The clinico-laboratory characteristics of groups I and II are shown in Table 2. Group II patients had a higher platelet count than group I patients (P < 0.001), but there were no other differences in parameters between the two groups. We determined 11 other gene mutations, including *FLT3-*ITD*, FLT3-*TKD*, CEBPA, MLL-*PTD*, KIT, JAK2, PTPN11*, *WT1*, *NRAS,* and *KRAS* mutations in patients with *NPM1* mutations*.* Group II patients had a higher frequency of *FLT3-*ITD. There was no association between immunophenotypic cluster and other gene mutations. The COOH-terminal nucleotide changes of *NPM1*gene were also checked, but not correlated to the immunophenotypic cluster.

**Table 2:** **Comparison of clinical-laboratory characteristics between cluster group I and II patients with *NPM1 *mutated AML**^**#**^

	**Cluster group I (n = 82)**	**Cluster group II (n = 12)**	**P-Value**
Age			1.000
Adult(>18 years)	81	12	
Children	1	0	
Gender			1.000
Male	38	6	
Female	44	6	
Laboratory data			
WBC(uL)	35710	43580	0.409
Hemoglobin (g/dL)	8.5	8.5	0.710
Platelet(uL)	49000	79000	<0.001
LDH (units/L)	1056	1253	0.803
FAB subtype			0.173
M0	0	0	
M1	17	0	
M2	26	7	
M3	0	0	
M4	28	5	
M5	10	0	
M6	1	0	
M7	0	0	
Undetermined	0	0	
Cytogenetic			0.662
Normal karyotype	67	10	
Abnormal karyotype	10	2	
Associated gene mutation*			
*FLT3-*ITD	36(44)	10(83)	0.013
*FLT3-*TKD	12(15)	1(8)	1.000
*NRAS*	9(11)	3(25)	0.179
*KRAS*	0	0	NA
*PTPN11*	7(9)	0	0.589
*KIT*	0	0	NA
*JAK2*	0	0	NA
*MLL-*PTD	0	0	NA
*WT1*	2(2)	0	1.000
*CEBPA*	4(4)	0	1.000
Immunophenotype**			
HLA-DR	34(41)	12(100)	<0.001
CD7	3(4)	12(100)	<0.001
CD13	76(93)	12(100)	1.000
CD14	16(20)	2(17)	1.000
CD15	36(47)	5(42)	1.000
CD33	82(100)	12(100)	1.000
CD34	9(11)	11(92)	<0.001
CD56	19(23)	0	0.117

Abbreviation: *ITD*, internal tandem duplication; *TKD*, tyrosine kinase domain mutation; *PTD*, partial tandem duplication; *NA*: not application.

^# ^Only 94 patients had complete immunophenotyping data and can be stratified by clustering analysis.

* Number of patients (% of patients with this gene mutation in each cluster group).

**Numbers of patients (% of patients with this antigen expression in each cluster group).

All four patients with CEBPA mutation were mono-allelic.

Ninety-four patients were recruited in cluster analysis of immunophenotype. There were 31 patients who received supportive care alone due to old age and frailty. Sixty-three patients were treated with standard intensive chemotherapy of idarubicin 12 mg/m^2^ per day on days 1–3 and cytarabine 100 mg/m^2^ per day on days 1–7 and then 2–4 courses of consolidation chemotherapy with high-dose cytarabine (2000 mg/m^2^ q12 h days 1–4, total 8 doses), with or without one anthracycline after complete remission (CR) was achieved. Eight patients died following induction chemotherapy, and eleven patients received HSCT and their disease free survival was censored at the time of transplantation.

**Figure 2:**
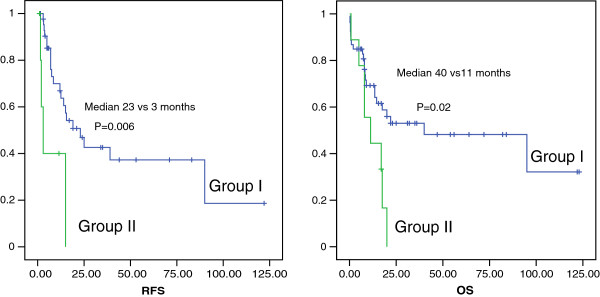
**Kaplan-Meier survival curves of relapse-free survival (RFS, left curve) and overall survival (OS, right curve) of 94 *NPM1- *mutated patients stratified by immunophenotypic clustering profile.**

With a median follow-up time of 53 months, the group II patients had a significantly shorter relapse-free survival (RFS; median, 3 vs. 23 months; p = 0.006; Figure 2) and overall survival (OS; median, 11 vs. 40 months; p = 0.02; Figure 2) than group I patients. For practicality, we compared the outcome of patients with positivity for all HLA-DR, CD34, and CD7 (n = 11) with that of other patients (n = 83); it was also shown that the former groups had a significantly worse prognosis than the latter group (RFS, *p* = 0.006; OS, *p* = 0.02).

We performed Cox regression multivariate analysis of variables, including clinico-laboratory data (age, gender, white blood cell count, hemoglobin level, platelet count, lactic dehydrogenase level, FAB subtype), cytogenetics, gene mutations, and immunophenotypic cluster (Table 3). The multivariate analysis revealed that immunophenotypic cluster was an independent prognostic factor (RFS, *p* < 0.001; OS, *p* = 0.001) in AML patients with *NPM1* mutations.

**Table 3:** **Multivariate cox regression of prognostic factors in *NPM1*-mutated patients**

**Factor**	**Relapse-free survival (RFS) p-value**	**Odd Ratio**	**95% CI**	**Overall survival (OS) p-value**	**Odd Ratio**	**95% CI**
Elderly(Age > 60 year)	0.015	0.933	0.056-15.647	0.629	NA	NA
Gender (Male vs Female)	0.558	NA	NA	0.871	NA	NA
Immunophenotypic cluster (Group II vs Group I)	<0.001	2.190	0.363-13.219	0.001	10.435	1.217-89.461
White blood cell count	0.371	NA	NA	0.898	NA	NA
Hemoglobin	0.836	NA	NA	0.349	NA	NA
Platelet	0.590	NA	NA	0.237	NA	NA
Lactate dehydrogenase	0.141	NA	NA	0.942	NA	NA
Cytogenetic* (Normokaryotype vs Additional changes)	0.597	NA	NA	0.464	NA	NA
CEBPA mutation (mutant vs wild type)	0.982	NA	NA	0.309	NA	NA
FLT3-ITD (mutant vs wild type)	<0.001	2.243	0.798-6.306	0.387	NA	NA
FLT3-TKD (mutant vs wild type)	0.191	NA	NA	0.135	NA	NA

*NA*, not applicable, Cytogenetics*: The cytogenetic data includes 91 patients with normal karyotype, 12 patients with additional changes, and 5 patients showed no mitosis.

### Hierarchical cluster analysis and survival analysis in validation cohort

In order to confirm the above finding of the prognostic value of immunophenotypic clusters, we collected another cohort of 36 AML patients with *NPM1* mutation diagnosed between 2008 and 2010. This validation cohort comprised 21 women and 15 men, with a median age of 59 years (range 18 to 84). The clinical characteristics were similar between these two cohorts, including age, gender, white blood cell count, hemoglobin level, platelet count, and serum lactate dehydrogenase level. Hierarchical cluster analysis also showed two distinct clusters. Group I patient showed significant better relapse free survival (median: 19.5 months vs. 10.5 months, p = 0.021), and overall survival (median: 32 months vs. 13 months, p = 0.055, Additional file 1: Figure S1). This finding confirmed that *NPM1-*mutated AML patients of immunophenotypic cluster group had worse prognosis.

**Figure 3:**
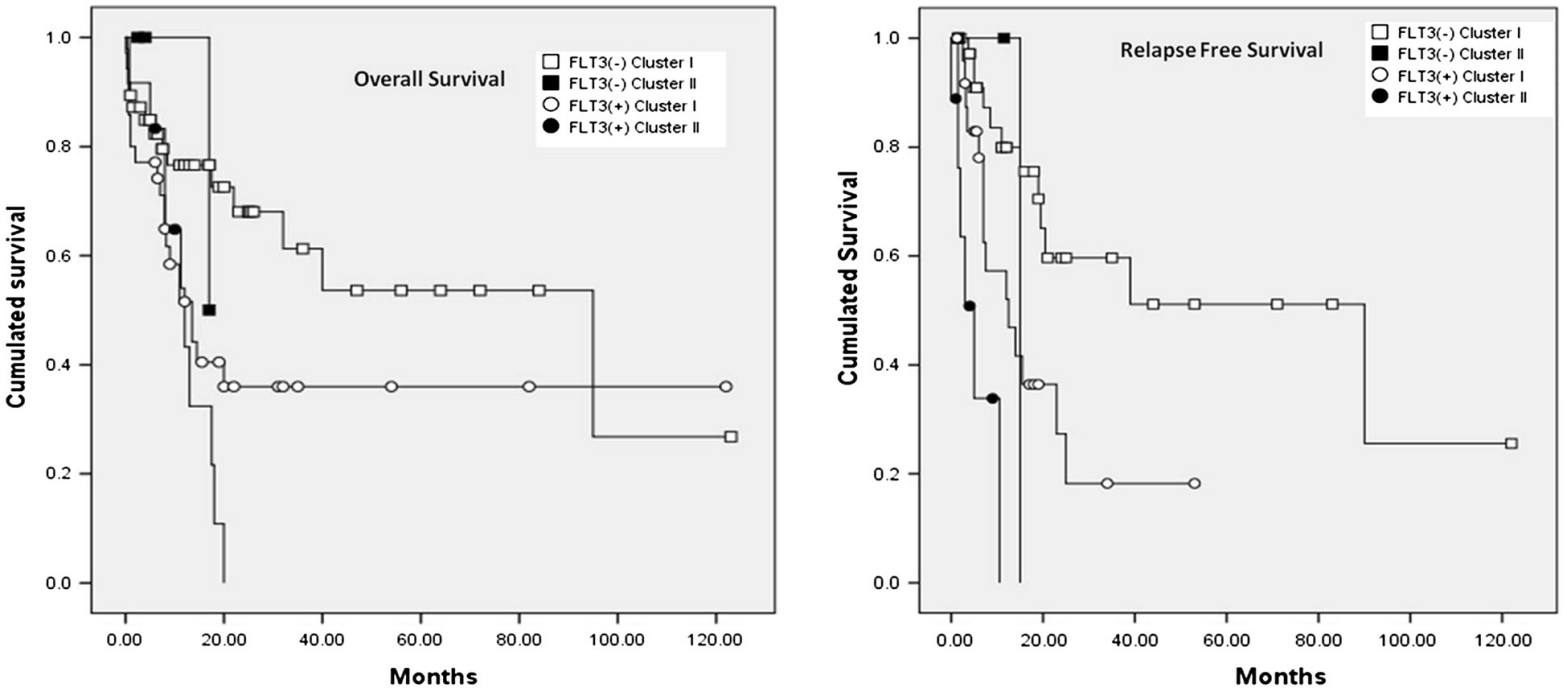
**Kaplan-Meier survival curves of relapse-free survival (left) of all 130 *NPM1- *mutated patients stratified by immunophenotypic clustering profile and FLT3 ITD mutation. **(p < 0.001). Kaplan-Meier survival curves of overall survival (right) of all 130 *NPM1- *mutated patients stratified by immunophenotypic clustering profile and FLT3 ITD mutation. (p = 0.017).

To further evaluate the significance of immunophenotypic cluster in the prognostic implication of *NPM1*-mutated AML, we incorporated both immunophenotypic cluster and the status of FLT3-ITD into survival analysis in a total 130 *NPM1*-mutated patients. Combined *FLT3-ITD* and immunophenotypic cluster could stratify these patients into distinct group (RFS, p < 0.001; OS, p = 0.017). The Kaplan-Meier survival curves of relapse free survival and overall survival are shown in Figure 3.

## Discussion

Immunophenotype analysis is highly helpful for the diagnosis and monitoring of minimal residual disease in hematologic malignancies [3-5]. Though leukemic blasts from *NPM1-*mutated AML usually show a specific immunophenotype with expression of CD13 and CD33 but absence of CD34 and HLA-DR [11], different expression patterns of surface markers on leukemic cells from individual patients are frequently seen [24]. In this cohort study, hierarchical cluster analysis revealed two distinct immunophenotypic clusters in *NPM1*-mutated patients. Most patients in group I showed CD7(−) CD33(+) CD34(−), while almost all patients in group II expressed HLA-DR, CD7, CD33, and CD34 on leukemic cells. The patients in immunophenotypic cluster group II had poorer outcomes than cluster group I, and the immunophenotypic cluster was an independent prognostic factor.

Some AML subtypes showed specific immunophenotypic patterns of leukemic cells, such as coexpression of CD15, CD34 and sometimes, CD19 in AML with t(8;21); coexpression of CD13, CD33 in absence of CD34 and HLA-DR in AML with t(15;17) [4-6]; and coexpression of CD34, HLA-DR, CD15 and CD7 in AML with *CEBPA* mutation [7]. The prognostic implication of immunophenotype in AML remains controversial [15,16]. For example, the negative prognostic effect of CD34 expression has been reported in some studies [25,26], but not in others [27,28]. The same is also true for CD7 expression. Most studies analyzed the correlation of the expression of a single marker with clinico-laboratory characteristics in a rather heterogeneous group of AML patients. In this study, the immunophenotypic cluster profiles were analyzed in a relatively homogeneous population of AML patients. Immunophenotypic cluster profiles provided distinct prognostic information in *NPM1*-mutated AML patients.

There are several large studies of *NPM1 mutation* in AML; the presence of *FLT3-ITD* is shown to be a poor prognostic factor in *NPM1*-mutated patients [29,30]. In order to clarify the association of the immunophenotypic patterns of *NPM1*-mutated AML with other gene mutations in AML, we checked class I (*FLT3*-ITD*, FLT3-*TKD*, PTPN11, JAK2, KIT, NRAS, KRAS,* and *WT1*) and class II gene mutations (*CEBPA* and *MLL-PTD*). Although the patients in immunophenotypic cluster group II had a higher incidence of *FLT3*-ITD, a mutation associated with poor prognosis [31], Cox regression multivariate analysis revealed that the immunophenotypic cluster was an independent prognostic factor (RFS, p < 0.001; OS, p = 0.001) in AML patients with *NPM1* mutations. To further evaluate whether the data of flow cytometry could be directly applied for prognostic prediction in clinical practice, we compared the survival between the patients with expression of all HLA-DR, CD34 and CD7 on leukemic cells and other patients. We found that positivity of all three markers was associated with shorter RFS and OS (*p* = 0.006 and 0.02, respectively). So, it may be worthwhile to use the expression pattern of these three antigens obtained from flow cytometry to predict the survival of patients at diagnosis.

Immunophenotypic cluster in *NPM1*-mutated AML patients has not been described before. In order to confirm the prognostic effect of the immunophenotypic cluster, we validated the correlation of immunophenotypic cluster and clinical outcome in another cohort of 36 *NPM1*-mutated patients diagnosed between 2008 and 2010. Hierarchical cluster analysis also showed two distinct clusters, group I patient showed significant better RFS (median: 19.5 vs. 10.5 months, p = 0.021) and OS (median: 32 months vs. 13 months, p = 0.055). This study was limited to a single university hospital, so the prognostic effect of immunophenotypic cluster should be further validated. Recently, *IDH1, IDH2* and *DNMT3A* mutations have also been reported in AML patients with *NPM1* mutation [32-34]. Correlating the immunophenotypic cluster with new biomarkers may also provide more insight into the molecular mechanisms of leukemogenesis in the future.

## Conclusions

In summary, hierarchical cluster analysis of the immunophenotypic profile was able to separate AML patients with *NPM1* gene mutations into two distinct groups with different prognosis. The immunophenotypic cluster with HLA-DR(+) CD34(+) CD7(+) is a poor prognostic factor independent of *FLT3-ITD* in AML patients with *NPM1* mutations.

## Competing interests

All authors declare they have no competing interests.

## Authors’ contributions

CYC, WCC, and HFT participated in the study design. CYC, SYH, WT, MY, JLT and HFT participated in data collection and analysis. CYC, and HFT participated in editing and proof reading. All authors read and approved the final manuscript.

## Pre-publication history

The pre-publication history for this paper can be accessed here:

http://www.biomedcentral.com/1471-2407/13/107/prepub

## Supplementary Material

Additional file 1: Figure S1 Kaplan-Meier survival curves of relapse-free survival (RFS, left curve) and overall survival (OS, right curve) of the validation cohort of 36 *NPM1- *mutated patients stratified by immunophenotypic clustering profile. Click here for file
