# Supplementary figures and images for: Hierarchical cluster analysis of immunophenotype classify AML patients with NPM1 gene mutation into two groups with distinct prognosis
Source: BMC Cancer. 2013 Mar 8;13:107. doi: 10.1186/1471-2407-13-107 (PMC3599624; doi:10.1186/1471-2407-13-107)

## Slide 1
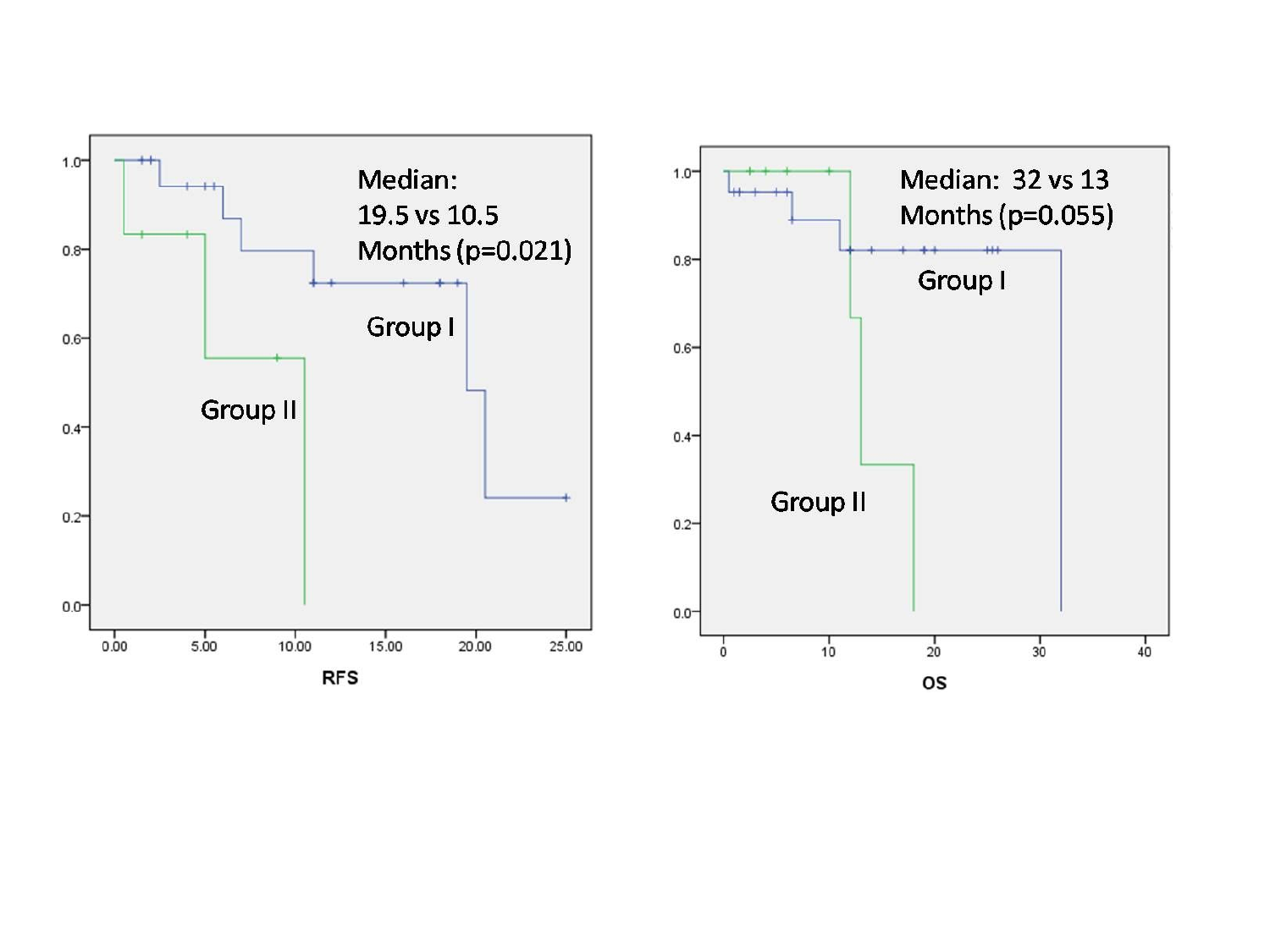

Supplement: Additional file 1: Figure S1 — Kaplan-Meier survival curves of relapse-free survival (RFS, left curve) and overall survival (OS, right curve) of the validation cohort of 36 NPM1- mutated patients stratified by immunophenotypic clustering profile. [file 1471-2407-13-107-S1.ppt]
